# Contribution to the Knowledge of *Leontodon* Sect. *Asterothrix* (Cass.) Ball in Italy and on the Balkan Peninsula

**DOI:** 10.3390/biology14091263

**Published:** 2025-09-12

**Authors:** Fabio Conti, Luca Bracchetti, Marco Dorfner, Ramona Schöpf, Nadine Benda, Christoph Oberprieler

**Affiliations:** 1 Scuola di Bioscienze e Medicina Veterinaria, Università di Camerino—Centro Ricerche Floristiche dell’Appennino, Parco Nazionale del Gran Sasso e Monti della Laga, S. Colombo, I-67021 Barisciano, L’Aquila, Italy; 2 Scuola di Bioscienze e Medicina Veterinaria, Unità di Ricerca e Didattica di San Benedetto del Tronto (URDIS), Università di Camerino, Via A. Scipioni 6, I-60074 San Benedetto del Tronto, Ascoli Piceno, Italy; luca.bracchetti@unicam.it; 3 Evolutionary and Systematic Botany Group, Institute of Plant Biology, University of Regensburg, Universitätsstr. 31, D-93053 Regensburg, Germany; marco.dorfner@ur.de (M.D.); ramona.schoepf@stud.uni-regensburg.de (R.S.); nadine.benda@stud.uni-regensburg.de (N.B.); christoph.oberprieler@ur.de (C.O.)

**Keywords:** AFLPseq fingerprinting, endemic, integrated taxonomy, vascular plants

## Abstract

In order to better understand the *Leontodon crispus* group within *L.* sect. *Asterothrix*, we collected specimens belonging to this group during several journeys to the Apennines and the Balkan Peninsula, especially to Croatia, Albania, and Greece. In the present contribution, *Leontodon biscutellifolius* is lectotypified; the *locus classicus* of this taxon is located in the Abruzzo Mts (central Italy). As a consequence, the taxon is confirmed for the Italian flora (central and southern Apennines) and a new combination on variety rank is proposed: *L. crispus* var. *biscutellifolius*. Plants from the Balkan Peninsula, currently named *L. biscutellifolius*, were compared from a morphological point of view with the Apennines populations. Balkan populations show denser indumentum on the leaves, usually consisting of 4-fid hair vs. usually 3-fid hairs in the Apennines. Additionally, the ray number of marginal hairs on the external involucral bracts was found to be 3–5 in the Apennines vs. 4–6 in the Balkan Peninsula accessions. Morphological variation was found to be in correspondence with a variation in genetic respects based on DNA-based fingerprinting (AFLPseq). The oldest subspecific name that can be used for Greek and Albanian populations is *L. crispus* subsp. *asper*. A morphologically and genetically deviating population from Southern Pindus (Greece) is described here as *L. hellenicus* subsp. *valeriae*.

## Simple Summary

*Leontodon biscutellifolius* is confirmed for the Italian flora (central and southern Apennines) and a new combination is proposed: *L. crispus* var. *biscutellifolius*. *Leontodon crispus* subsp. *asper* occurs on the Balkan Peninsula. A morphologically and genetically deviating population from Southern Pindus is described as *L. hellenicus* subsp. *valeriae*.

## 1. Introduction

The genus *Leontodon* L. (*Asteraceae*, tribe *Cichorieae*) comprises over 50 taxa predominantly distributed throughout Europe and the Mediterranean basin [1]. Its section *L.* sect. *Asterothrix* (Cass.) Ball is distributed across the Mediterranean basin, extending from the Iberian Peninsula to southwestern Asia and includes 21 species [2]. It is characterized by leaves and phyllaries bearing three- to multiple-fid hairs, and by all achenes being equipped with pappus bristles.

According to Zidorn [3], *L.* sect. *Asterothrix* represents the most complex clade within the genus; in particular, the taxonomic delimitation of *L. crispus* Vill. *s.lat.* remains contentious. Samuel et al. [4] conducted a molecular phylogenetic analysis of the genus and corroborated the distinctiveness of *L.* sect. *Asterothrix*.

Due to the difficulties in identifying the taxa belonging to this section, and in order to contribute to the knowledge of this taxonomically critical group in recent years, we collected some specimens of *Leontodon* during several journeys to the Apennines and the Balkan Peninsula, in Croatia, Albania, and Greece.

We were interested in clarifying what the name *L. biscutellifolius* relates to and defining its distribution of this taxon. Currently, it is reported for Southeastern Europe to Turkey, Caucasus, and European Russia, but it is excluded from the Italian flora [1,2,3,5]. The closest species is *L. crispus* Vill., a southern European species (France, Spain, Italy, Austria, Helvetia, and the Balkan Peninsula). In Italy, *L. crispus* is known from the Alps and the Apennines [6]. The two taxa are recognizable mainly based on characteristics of the margin of external involucral bracts, namely being pectinate-ciliate with 5–7-fid white hairs in *L. biscutellifolius* and absent or reduced to shorter simple (occasionally 2–3-fid) hairs in *L. crispus* [5]. In central and southern Italy, some authors reported on *Leontodon crispus* subsp. *asper* (Waldst. & Kit.) Rohlena (currently *L. biscutellifolius*) in the past [7,8,9,10,11,12,13]; however, according to Pittoni [5] and Zidorn [3], these populations do not belong to *L. biscutellifolius* as occurring on the Balkan Peninsula. Based on pappus length and leaf shape, these populations were assigned to *L. crispus*. According to our own observations, plants from the Apennines have involucral bracts with pectinate, 2–5-fid hairs. As a consequence, we initiated and performed a morphological and molecular comparison of *L. crispus*, *L. biscutellifolius s.lat.*, *L. hellenicus*, *L. incanus*, *L. albanicus* subsp. *albanicus*, *L. albanicus* subsp. *acroceraunicus*, and a new population found on Southern Pindus (Greece). While the comparison between *L. albanicus* subsp. *albanicus* and *L. albanicus* subsp. *acroceraunicus* was recently published [14], we here report on the findings of these newly gained insights into the remainder of the study group.

## 2. Materials and Methods

The present study is based on field surveys, an extensive analysis of the relevant literature, and an examination of herbarium specimens of *L. biscutellifolius* and *L. crispus* kept in APP, BP, CAME, FI, G, JE, NAP, PR, and RO (acronyms following [15]) occurring in southern Europe. Samples housed in APP were collected mainly by one of the authors (F.C.), starting in 1991. Additional explorations have been conducted over the years, specifically in 2012, 2015, 2018, 2023, and 2024, trying to understand the variability of the populations located at sites such as Çika, Nemercka, Tzoumerka, Timfristos, Kaliakouda, and Mt. Chelidon (see list of speciminens examined).

The protologues of the entities belonging to the investigated group were searched and consulted.

Morphometric analysis. Taxonomically significant morphological traits of *Leontodon* (refer to Table 1) were examined and quantified using a stereoscopic microscope and a digital caliper calibrated to 0.1 mm precision.

A total of 28 morphological characters were selected and scored for 15 herbarium specimens of *L. crispus s.str.*, 17 of *L. biscutellifolius* from Central Southern Apennines (Lazio, Abruzzo, Molise, Campania, Basilicata), 31 of *L. crispus* subsp. *asper* from the Balkan Peninsula (Montenegro, Albania, Greece), 20 of *L. hellenicus*, and 22 of an atypical *L. hellenicus* from Southern Pindus. This resulted in a dataset of 105 individuals and 28 variables, to which 24 herbarium specimens of *L. albanicus* subsp. *albanicus* and 15 of *L. albanicus* subsp. *acroceraunicus* were added, which have already been analyzed in Conti et al. [14]. Among the variables examined, 20 were quantitative continuous and 8 were quantitative discrete (Table 1).

In order to compare the five considered groups, multivariate analysis via MANCOVA test [16] was performed. To simplify the dataset complexity and reveal underlying patterns of character correlations, the data were then subjected to a second multivariate analysis using Spearman Principal Component Analysis [17,18]. Moreover, mean and median values, 25th and 75th percentiles, and standard deviations were calculated, and box plots were generated for the five groups and each variable. In the descriptions of the taxonomic treatment, percentiles are reported and extreme values are mentioned in brackets. The software program XLSTAT version 2024 [19] was used to carry out these analyses.

**Figure A1:**
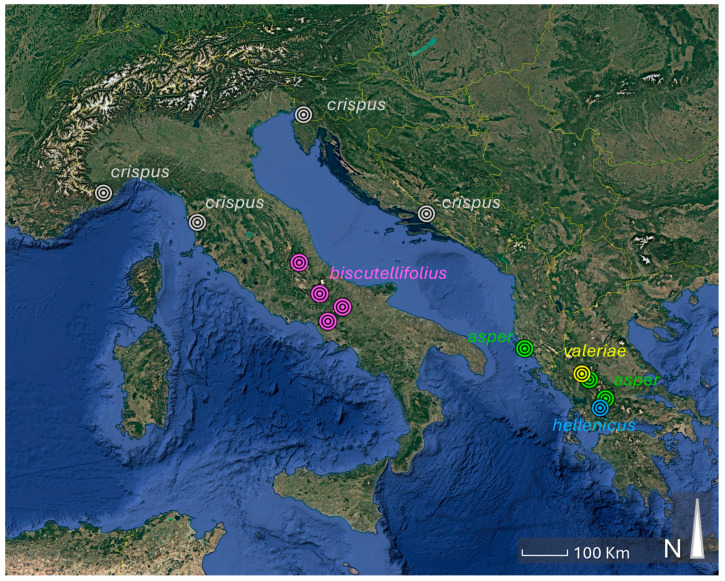
Map of studied accessions.

DNA extraction and AFLPseq fingerprinting—The groups identified on a morphological basis were selected and analyzed from a molecular point of view. Genomic DNA for genetic fingerprinting was extracted according to the CTAB DNA extraction protocol of Doyle and Dickson [20] and Doyle and Doyle [21]. The protocol for the recently described AFLPseq fingerprinting procedure [22] followed exactly the previously described wet lab and bioinformatical procedures designed for *Leontodon* accessions (see [14]). The accessions used in the present study are listed in the Appendix A (Table A1), mapped in Figure A1, and all came from material housed in the *Herbarium Apenninicum* (APP).

Read data processing, de novo locus assembly, identification of orthologous loci, reference-based SNP calling with the SLANG pipeline, and the final calculation of frequency-sensitive SNP-based Jukes–Cantor (JC) distances followed the protocol described by Dorfner et al. [22] and our previous *Leontodon* study [14]. Based on these pair-wise JC distances, a phylogenetic network reconstruction using the NeighborNet method in SPLITSTREE v.4.16.1 [23] was carried out.

## 3. Results

**Figure 1:**
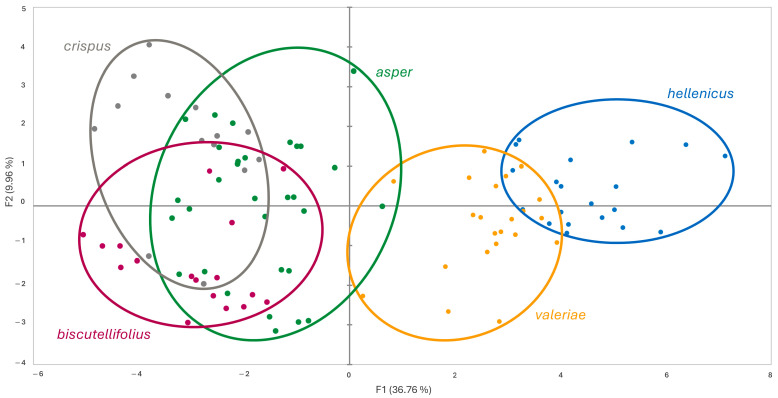
Scatter plot of Principal Component Analysis based on morphological data for all studied groups.

The comparison of the five studied population groups (*L. hellenicus* from its currently known distribution area, *L. hellenicus* from southern Pindus, *L. crispus* subsp. *cripus*, *L. crispus* subsp. *asper* from the Balkan Peninsula, and *L. biscutellifolius* from central-southern Apennines) was carried out using Manova analysis (Wilks’ test, Rao’s approximation); its null hypothesis states that each group is similar to each other. Based on the resulting *p*-values (<0.0001 with F = 22.212), it was possible to highlight significant differences among groups. Principal Component Analysis (PCA) was then performed to explore possible patterns responsible for their morphological distinctness. The two principal factors accounted for 46.72% of the total variance (F1 = 36.76%, F2 = 9.96%), and the first one provided an appreciable separation between two OTU groups: on the left, accessions of *L. hellenicus* and representatives of this taxon from Southern Pindus are found, while on the right, specimens of *L. crispus* subsp. *crispus*, *L. crispus* subsp. *asper*, and *L. biscutellifolius* cluster together. In the former cluster, the separation between the two taxa appears very marked (Figure 1). Based on the generated squared cosines table, the variables that contributed most to the differentiation were as follows: for Factor 1, plant height (0.62), leaf length (0.4), width of leaf teeth (0.51), number of hairs per 1 mm^2^ on leaf surface (0.8), leaf hairs: most common number of distal rays (0.72), minimum number of distal rays (0.81), maximum number of distal rays (0.73), stalk length (0.73), maximum stalk length (0.72), hairs on external involucral bracts: hair length (0.37), fid hairs on external involucral bract margins: ray length (0.53), fid hairs on middle involucral bract: number of rays (0.36), ray length (0.56), pappus plumes’ length (0.3); and for Factor 2, hairs on external involucral bracts: hair length (0.71) and fid hairs on middle involucral bract: stalk length (0.55).

**Figure 2:**
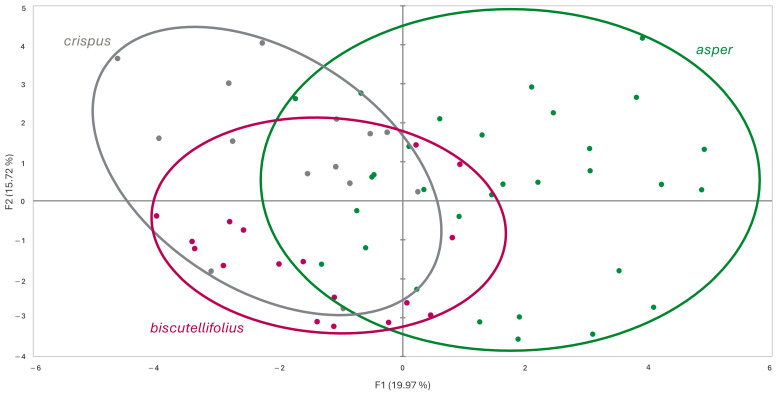
Scatter plot of Principal Component Analysis based on morphological data for *L. crispus* subsp. *asper*, *L. crispus* subsp. *crispus* var. *crispus*, and *L. crispus* subsp. *crispus* var. *biscutellifolius*.

To investigate the resulting second grouping, further PCA was performed (F1 = 19.97%, F2 = 15.72%); the scatter plot (Figure 2) showed a differentiation of *L. crispus* subsp. *crispus* from *L. crispus* subsp. *asper* and *L. biscutellifolius*. The variables’ contribution were as follows: for Factor 1, fid hairs on middle involucral bract: stalk length (0.3), leaf length (0.49), leaf width (0.38), width of leaf teeth (0.31), number of hairs per 1 mm^2^ on leaf surface (0.64), leaf hairs: most common number of distal rays (0.48), minimum number of distal rays (0.49), maximum number of distal rays (0.29); and for Factor 2, fid hairs on external involucral bracts: number of rays (0.62), hairs on external involucral bracts: hair length (0.64), fid hairs on middle involucral bract: number of rays (0.6), stalk length (0.4), and ray length (0.53).

Additionally to our morphometric and genetic results, we confirm the presence of *L. biscutellifolius* in Italy, its *locus classicus*. It is endemic to the central and southern Apennines. From a morphological comparison of *L. biscutellifolius* s.l. between Apennines and Balkan populations (*L. crispus* subsp. *asper*) with the typical *L. crispus* from Alps, northern Apennines and Croatia, it emerges that it is possible to distinguish two groups for different geographical areas by subspecific rank: *L. crispus* subsp. *crispus* and *L. crispus* subsp. *asper*. *Leontodon biscutellifolius* is not distinguished from *L. crispus s.str.*, except by having ciliated outer bracts with fid hairs vs. simple or absent hair on the margins, and the diversity is not even confirmed by molecular analyses (see below). Therefore, we propose to recognize it at variety rank: *Leontodon crispus* var. *biscutellifolius*. Balkan populations show a denser indument on the leaves, usually 4-fid hair vs. usually 3-fid hair in the Apennines and external bract margin hairs’ number of rays, mainly 3–5 vs. 4–6. *Leontodon crispus* subsp. *asper* is variable and several taxa have been described that deserve further investigation such as *L. haussknechtii* Uechtr. ex Hausskn. or *L. crispus* var. *setulosus* (Halácsy) Kupicha, *L. crispus* subsp. *rossianus* (Degen & Lengyel) Hayek, or *L. crispus* subsp. *saxatilis* (Ten.) Nyman. As a consequence, we herewith propose only a provisional taxonomic treatment of the study group. The population of the southern Pindus included in our present analysis is close to *L. hellenicus* and shows a peculiar feature combination in morphological respect together with a strong genetic distinctness from *L. hellenicus s.str.* (see below). Therefore, we describe it as a subspecies new to science named *L. hellenicus* subsp. *valeriae.*

**Figure 3:**
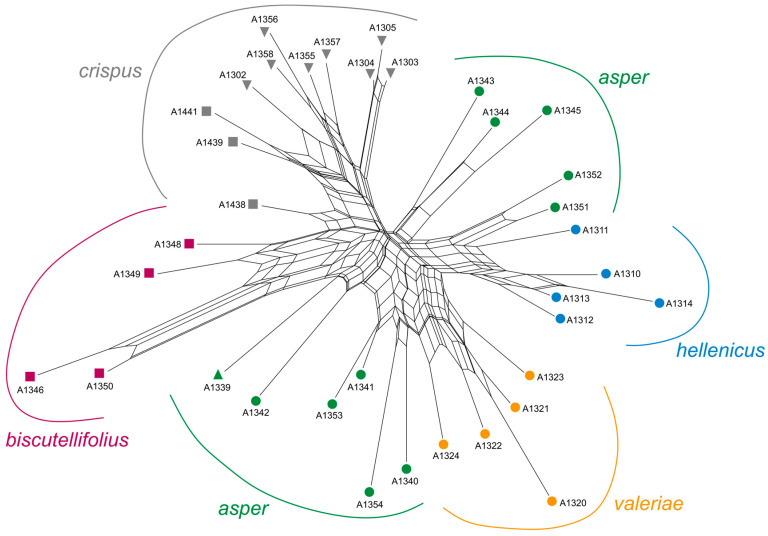
NeighborNet network of 36 accessions of *Leontodon* sect. *Asterothrix* based on 12,183 SNPs from 2681 loci resulting from AFLPseq fingerprinting with Jukes–Cantor distances as a measure of genetic similarity among accessions. While colors signify taxa, geographical information is depicted as shape differences (i.e., squares: Italy; down-pointing triangles: Croatia; upwards-pointing triangles: Albania; circles: Greece).

*AFLPseq fingerprinting*—In total, 449,762 reads and 165.91 Mbp were sequenced for the 36 *Leontodon* accessions of the present study. After read preprocessing, 344,518 reads (74.95 Mbp) with lengths between 50 bp and 700 bp passed the Q5 quality filter. With the SLANG pipeline (cluster thresholds optimized to values of 0.85), 2681 orthologous loci were inferred, containing 12,183 SNPs. After calculation of pair-wise Jukes–Cantor (JC) distances based on the SNP matrix, the NeighborNet network depicted in Figure 3 was received. Despite considerable signals of reticulation seen in this graphic representation of genetic relationships, a non-accidental geographical pattern emerges with accessions of the taxa *crispus* and *biscutellifolius* from Italy (squares) and Croatia (down-pointing triangles) on one side and accessions of the taxa *hellenicus* and *valeriae* from Greece (dots) on the other side. This clear bipartition, however, receives considerable fuzziness by accessions assigned to the taxon *asper* from Albania (upwards-pointing triangle) and Greece (green dots). Additionally, many of these are also found in mediating positions to the taxa *biscutellifolius* (A1339 and A1342), *valeriae* (A1340 and A1354), or *hellenicus* (A1351 and A1352). An intermediate position is also observed for accession A1438 from Monte Vaso (Italy, Tuscany region, Pisa province), geographically mediating between *crispus* accessions from further north (A1439: Trieste province; A1441: Imperia province) and those from central (A1348: Teramo province; A1349: L’Aquila province) and southern Italy (A1346: Caserta province; A1350: Potenza province).

## 4. Discussion and Taxonomic Treatment

In both analyses, the one addressing morphology and the other based on AFLPseq fingerprinting, the majority of surveyed individuals are found being assigned to two poles of the total variation, with OTUs representing *crispus*/*biscutellifolius* on the one side and *hellenicus*/*valeriae* on the other. Without OTUs from the Balkan Peninsula of *asper*, these two poles would be completely and correspondingly separated from each other in both datasets. While in the molecular analysis, the accessions of *asper* hold an intermediate position between these two poles, the multivariate statistical analyses of morphological data demonstrate the closeness of these accessions to the *crispus*/*biscutellifolius* side.

Therefore, two options appear appropriate to account for the presently demonstrated variation of *L. crispus* on the Apennines and Balkan Peninsulas: (a) Acknowledgement of the *crispus*/*biscutellifolius* and the *hellenicus*/*valeriae* poles as two subspecies of *L. crispus*, with *asper* individuals connecting these two extremes as intermediates without any taxonomic rank. As a consequence, *crispus* and *biscutellifolius* on the one hand and *hellenicus* and *valeriae* would be mere varieties of these two subspecies then; (b) Acknowledgement of the *crispus*/*biscutellifolius* and the *hellenicus*/*valeriae* poles as two independent species and *asper* as the clinally mediating taxon between these two species. Owing to the morphological closeness of *asper* to the *crispus*/*biscutellifolius* pole and due to the demand to keep nomenclatural changes at a minimum until a final taxonomic treatment of *L. crispus* on the Balkan Peninsula, we here propose the treatment of *asper* as a subspecies of *L. crispus*, *crispus*/*biscutellifolius* as two varieties of *L. crispus* subsp. *crispus*, and *hellenicus*/*valeriae* as two subspecies of *L. hellenicus.*

***Leontodon crispus*** Vill., Prosp. Hist. Pl. Dauphiné: 34 (1779) subsp. ***crispus***

Protologue citation: no localities are reported.

Lectotype (designated by Talavera et al. [24] (p. 3611): France, “des Alpes voisines de Grenoble”, 1780, *D. Villars* (MPU No. 023657).

***Leontodon crispus*** subsp. ***crispus*** var. ***biscutellifolius*** (DC.) F. Conti, **comb. nov.**

≡ *Leontodon biscutellifolius* DC. in Prodr. 7: 103 (1838)

≡ *Leontodon asper* var. *biscutellifolius* (DC.) Boiss., Fl. Orient. 3: 730 (1875)

Protologue citation: In pascuis siccis Apennini Neapolitani (Ten!) et Romani (Mor!), in Tauriae herbidis (Bieb. Stev.!).

**Lectotype (designated here)**: Italy. *Apargia macrorhiza* Gant.?/In Aprutio, *M. Tenore* (G-DC No. SIB 347247/1, reçu en 1833, digital image!).

Typification notes: We traced two herbarium sheets in G-DC. One of them, with several individuals mounted on the sheet, the left one bearing a label handwritten by M. Tenore: *Apargia macrorhiza* Gant.?/In Aprutio and the right one collected by Moricand in Mt. di Somma—Rome.

Aprutio, now Abruzzo, was the northernmost region of Napoli Kingdom.

The second sheet was collected by Steven in 1820 on Taur (Crimea) and it is certainly part of the original material used by De Candolle for the description of the species.

The first sheet and the left-hand specimen collected from the type locality by Tenore and housed in G (No. SIB 347247/1), which agrees with the protologue [25] and is in better conditions, is selected here as the lectotype.

***Leontodon crispus*** subsp. ***asper*** (Waldst. & Kit.) Rohlena in Sitzungsber. Königl. Böhm. Ges. Wiss. Prag, Math.-Naturwiss. Cl. 1911(1): 67. 1912

≡ *Apargia aspera* Waldst. & Kit., Descr. Icon. Pl. Hung. 2: 114. 1803

Protologue citation: “Crescit in sylvis ad thermas Herculis locis lipidosis”, currently Băile Herculane (Romania).

Lectotype (designated by Kovats [26] (pp. 33–53):—*Apargia aspera*, Herbar. Kitaibel., N. 113, Mus. Nat. Hung. fasc. XXVI (BP, No. 7024, digital image!)

≡ *Leontodon asper* (Waldst. & Kit.) Poir. in Lamarck, Encycl., Suppl. 3: 453. 1814 [non *Leontodon asper* Forssk. 1775]: it is based on a later homonym and therefore the name is illegitimate.

***Leontodon hellenicus*** Phitos, in Österr. Bot. Z. 113: 272. 1966 subsp. ***hellenicus***

Protologue citation: Prov. Evrytania: In declivibus.borealibus montis Caliacuda, circiter 1800 m.

Holotype:—Prov. Evrytania: In declivibus.borealibus montis Caliacuda, ca 1800 m, 24 June 1969, *D. Phitos 4394* (W No. 22548, digital image!).

**Figure 4:**
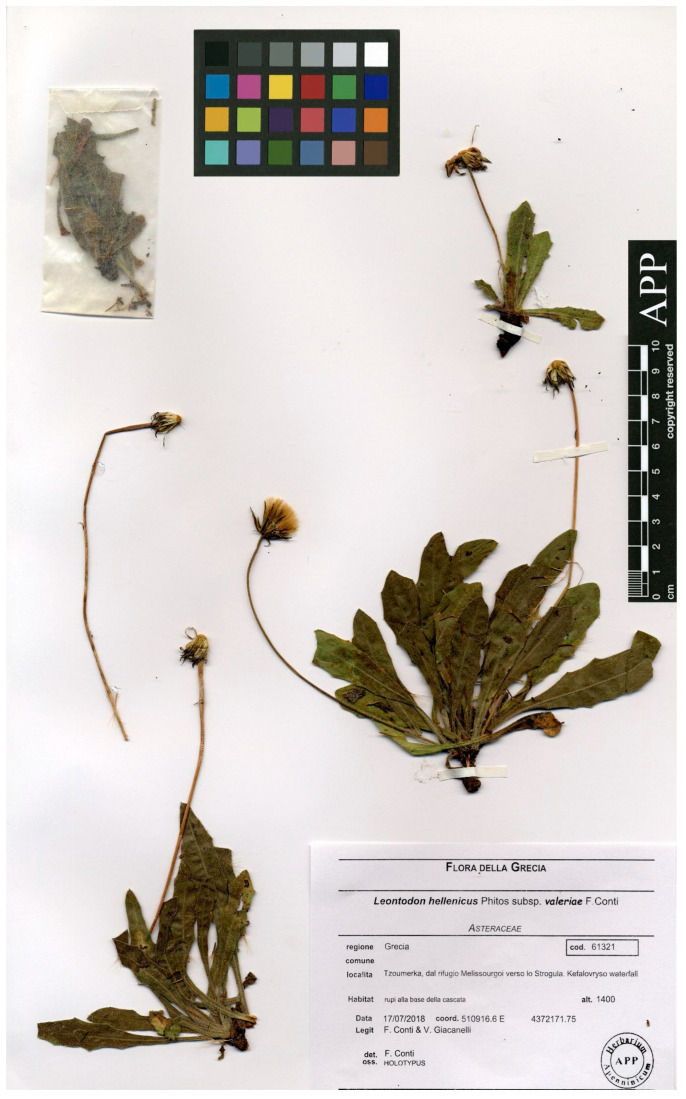
*Holotypus* of *Leontodon hellenicus* subsp. *valeriae* housed in APP.

**Figure 5:**
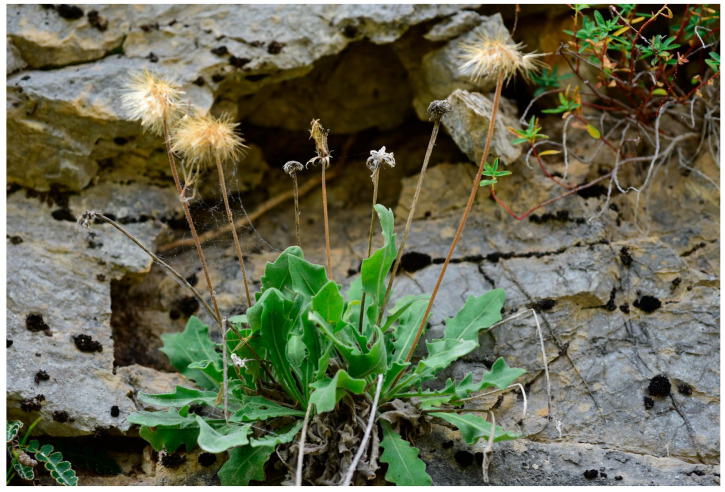
Fruiting individual of *Leontodon hellenicus* subsp. *valeriae* (photo by F. Conti).

**Figure 6:**
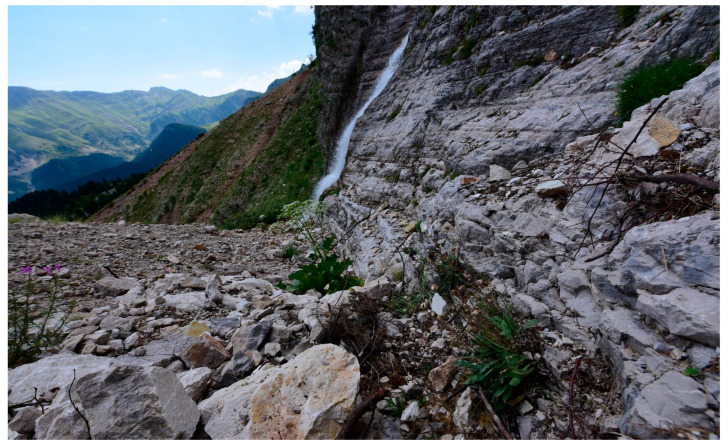
*Leontodon hellenicus* subsp. *valeriae* in its habitat and at the *locus classicus* (photo by F. Conti).

***Leontodon hellenicus*** subsp. ***valeriae*** F.Conti, **subsp. nov.** (Figure 4, Figure 5 and Figure 6)

Holotype: Greece. Pindo Sud, Tsoumerka, dal Rifugio Melissourgoi verso lo Strogula, cascate Kefalovryso, rupi alla base della cascata, 510916.6 E 4372171.75, 17/07/2028, *F. Conti*, *V. Giacanelli* (APP No. 61321) Isotypes (APP Nos. 61317, 61318, 61319, 61320).

Diagnosis: It is distinguished from *L. hellenicus* subsp. *hellenicus* by leaves with (12–) 17.2–27 (–41) hairs in 1 mm^2^ vs. (18–) 28.7–38 (–62); stalk length (0.1–) 0.13–0.2 (–0.27) [to maximally (0.16–) 0.24–0.38 (–0.46)] vs. (0.09–) 0.11–0.14 (–0.2) [to maximally (0.12–) 0.14–0.2 (–0.24)]; flower length (19–) 19.5–28.25 (–30) mm vs. (14–) 15.6–19 (–22) mm; and pappus length (8.5–) 9.0–10.87 (–11.5) mm vs. (6–) 7–8.8 (–10) mm.

Description: Perennial with a taproot and 1 unbranched stem, (72–) 117–164.2 (–195) mm tall. Stem ribbed with short, stalked hairs (3–) 4–7-fid hairs; bracts 0–3. Basal leaves rosulate (47.0–) 60.2–82.0 (–107.0) × (9.5–) 11–15 (–20) mm, oblanceolate, sinuate-dentate, with (3–) 4–6 (–7) teeth, teeth width (1–) 1.5–2.1 (–3.0) mm, greyish-green, with dense (number of hairs in 1 mm^2^) (12–) 17.2–27 (–41) , stalked usually 5–7.7 (–8)-fid hairs on both surfaces, min. 3–4 (–5) , max. (6–) 8–10 (–12)-fid hairs; stalk length (0.1–) 0.13–0.20 (–0.27) mm, max stalk length (0.16–) 0.24–0.38 (–0.46) mm; ray length (0.10–) 0.18–0.23 (–0.28) mm. Capitulum solitary. Involucre length (12–) 12.5–14.2 (–16) mm, linear-lanceolate phyllaries, in several rows, outer (3–) 4–5 (–7) mm long, with stalked 0–4 (–7)-fid hairs, 0–0.47 (–1.0) mm long on the surface, with moniliforms hairs (0–) 0.11–0.15 (–0.2) mm long, marginally pectinate-ciliate with fid hairs (2–) 3–4 (–9) -fid, stalk (0.02–) 0.06–0.17 (–0.5) mm long, rays (0.08–) 0.15–0.20 (–0.3) mm long. Middle bract surface fid hairs 0–3.75 (–5) -fid, stalk (0–) 0.01–0.28 (–0.55) mm long, rays (0–) 0.02–0.15 (–0.2) mm long. Ligules yellow (19–) 19.5–28.2 (–30) mm long. Achene (5–) 5.9–7.3 (–8) mm with triangular scale-like above, narrowed towards apex. Off-white pappus (8.5–) 9.0–10.9 (–11.5) mm, scabrid with few longer hairs 0–0.32 (–0.46) mm, denticules (0.04–) 0.05–0.07 (–0.08) mm. Flowering in June, fruiting in July.

Etymology: The specific epithet refers to Valeria, my companion who also followed me in the exploration of southern Pindus.

Distribution and habitat: It is known in Greece, from southern Pindus on Mt. Tzoumerka on vertical cliffs.

**List of the specimens examined**

***L. crispus*** subsp. ***crispus*** var. ***crispus***

Forti Civezzano (Trento), 18 July 1962, *F. Pedrotti s.n.* (CAME); ex pascuis Catria al Caprile, *s.d.*, *s.c.*, *s.n.* (RO); pascolo semisassoso nel Kiljas pr. Lussinpiccolo sul Quarnero, a 15 m, a.s.m., 19 April 1934, *G. Lusina s.n.* (RO); incolti aridi presso Lussinpiccolo nel Quarnero, 25 m a.s.m., 28 April 1934, *G. Lusina s.n.* (RO); fessure rocce sul telegrafo al sole, 80 m a.s.l., pr. Lussinpiccolo nel Quarnaro, *s.d.*, *G. Lusina s.n* (RO); Loc. Venetia, prov. Di Verona: in pascuis collium supra Torri del Benaco, solo calcareo, alt. 100–300 m (sed ascendit in M. Baldo usque ad 1000 m), Jun, Jul 1904, *G. Rigo s.n.* (RO); Loc. Venetia, Prov. Di Udine: Carnia, prope Tolmezzo, in declivibus mer. montis Strabut, loco dicto Pra’ Castello, locis herbosis et saxosis siccis, solo calcareo-dolomitico, alt. 380–480 m, 6 Jun 1905, *L. et M. Gortani*, *s.n.* (RO); […] tergestina […], *s.d.*, *Tommasini s.n.* (RO); Susa (alla Blanie), 10 Jun 1859, *s.c.*, *s.n*. (RO); Susa alla Blanie, 21 Mai 1863, *s.c.*, *s.n.* (RO); Alemanno presso il Brembo, *Cesati*, *s.n.* (RO).

***L. crispus*** subsp. ***crispus*** var. ***biscutellifolius***

ITALIA: Monte di Civitella del Tronto (Civitella del Tronto, TE), 17 June 1873, *D’Amato s.n.* (APP No. 6056); Gobbe di Selva Romana (Pennapiedimonte, CH), Pascoli aridi, 1800 m, 28 July 1991, *F. Conti s.n.* (APP No. 12988); Primo Circo glaciale sotto La Metuccia (Pizzone, IS), pendio arido, 1900 m, 20 July 1991, *F. Conti s.n.* (APP No. 22501); Morrone delle Rose (S. Biagio Saracinisco, FR), pendio arido, 1250 m, 2 October 1991, *F. Conti s.n.*(APP No. 22504); M. della Rocchetta (Castel S. Vincenzo, IS), prato arido, 900 m, 3 October 1991, *F. Conti s.n.* (APP No. 22891); Valle dell’Altare (Pizzone, IS), prato arido, 1600 m, 9 July 1992, *F. Conti s.n.* (APP No. 22892); Monte Rocchetta- propaggini settentrionali (Rocchetta a Volturno, IS), pendio arido, 700–750 m, 28 May 1993, *F. Conti s.n.* (APP No. 22498); Valle Fredda sopra il limite del bosco (Pizzone, IS), pendio arido, 1900 m, 6 July 1993, *F. Conti s.n.* (APP Nos. 22499, 22502); M. Argatone—Serra della Terratta (Bisegna, AQ), rupi e praterie altitudinali, 1900–2200 m, 1 August 1996, *F. Conti s.n.* (APP No. 40493); Monte Serrone (Campoli Appennino, LT), praterie altitudinali—pendii rupestri, 1800–1980 m, 31 July 1997, *F. Conti*, *F. Minutillo s.n.* (APP No. 39924); M. Viglio, sopra Meta (Civitella Roveto, AQ), pascoli, 1600–2150 m, 12 July 1998, *F. Conti s.n.* (APP Nos. 52267, 52291); V.ne della Terratta (Scanno, AQ), 13 July 2000, *F. Conti s.n.* (APP No. 49737); Monte Porrara presso la cima (Campo di Giove—Palena, AQ—CH), Pascoli, pendii rupestri, 1943 m, 20 June 2003, *F. Conti* et al. *s.n.* (APP No. 6631); Bocca Mezzana (Anversa degli Abruzzi, AQ), pascoli, 1450–1500 m, 15 June 2003, *D. Tinti s.n.* (APP No. 16427); Camosciara, Cascata delle Ninfe (Civitella Alfedena, AQ), 29 July 2004, *F. Conti*, *F. Bartolucci s.n.* (APP No. 35320); dal Rifugio del Monte al Vallone del Monte (Fano Adriano, TE), rupi (calcari marnosi e calcareniti) e pascoli su sedimenti morenici, 1615–2000 m, 19 June 2010, *F. Conti*, *F. Bartolucci*, *N. Ranalli s.n.* (APP No. 43736); Tra M. Irto e M. S. Nicola, Serra delle Gravare (Opi, AQ), 6 August 2010, *F. Conti*, *G. Serafini s.n.* (APP No. 48216); Faresano (Brienza, PZ), prato arido e gariga, 1050 m, 5 June 2013, *F. Conti*, *F. Bartolucci s.n.* (APP No. 52321); Madonna di Fradeianne, dalla deviazione per Pizzo San Salvatore a Madonna di Fradeianne (Pietramelara, CE), pascoli aridi, 890–925 m, 5 May 2017, *F. Conti*, *F. Bartolucci*, *R. Pennesi s.n.* (APP No. 58126); crinale del vallone ovest di Costa della Tavola (Rocca di Mezzo, AQ), parte sommitale di ghiaione, 2094 m, 25 July 2023, *F. Conti*, *G. Cangelmi s.n.* (APP Nos. 67601, 67602, 67603).

***L. crispus*** subsp. ***asper***

BOSNIA-HERZEGOVINA: Parco Naturale Konjuh, lungo la strada per Muska Voda scarpata lungo strada, 670 m, 7 June 2017, *F. Conti*, *C. Gangale*, *D. Uzunov s.n.* (APP)*;* Parco Naturale Konjuh, prato, 766 m, 7 June 2017, *F. Conti*, *C. Gangale*, *D. Uzunov s.n.* (APP).

MONTENEGRO: Canyon del fiume Tara, da Djurdievica Tara a Crna Poda, substrato calcareo, 600 m, 10 July 1996, *D. Lakušić*, *F. Conti*, *G. Tomović s.n.* (APP No. 8980); Mt. Prokletije, Bukumirsko jezero, bottom of Crvena Greda limestone, 18 July 2003, *D. Lakušić*, *F. Conti*, *Z. Bulić*, *M. Niketić*, *G. Ciaschetti*, *G. Tomović*, *S. Adžiablahović s.n.* (APP No. 31882); Mt. Prokletije, Popadija—Zuto prlo *Festuco-Seslerietea*, limestone, 1900 m, 18 July 2003, *D. Lakušić*, *F. Conti*, *Z. Bulić*, *M. Niketić*, *G. Ciaschetti*, *G. Tomović*, *S. Adžiablahović s.n.* (APP No. 31896);

ALBANIA: Nemercka (tra Permet e Kaluth), fascia montana 12July 2012, *F. Conti*, *M. Manilla s.n.* (APP No. 49009); Çika, vetta principale salendo dal passo Llogarasa 1700–2000 m, 9 July 2012, *F. Conti*, *M. Manilla s.n.* (APP Nos. 49303, 49333, 49385); Mali J Gier sopra Lefterochori Pendii rupestri, 1000–1200 m, 24 June 2015, *F. Conti*, *A. Stinca*, *R. Pennesi s.n.* (APP Nos. 56356, 56383); M. Çika tra Llogara e la vetta Pendii rupestri, 1020–1990 m, 23 June 2015, *F. Conti*, *D. Lakušić*, *R. Di Pietro*, *N. Kuzmanović*, *A. Stinca*, *S. Đurović*, *I. Janković*, *R. Pennesi s.n.* (APP Nos. 56425, 56476); Presso Fushëbardhë Pendii rupestri e pascoli, 570–670 m, 27 June 2015, *F. Conti*, *R. Di Pietro*, *A. Stinca*, *R. Pennesi s.n.* (APP No. 57133).

GREECE: Pindo sud sopra Vradeto, pascoli, 1500–1800 m, 16June 1998, *F. Conti s.n.*(APP No. 47787); M.Tsoumerka-Pindo sud presso Melissourgoi Vers: N, 1300 ca. m, 14 June 1998, *F. Conti s.n.* (APP No. 47847); M. Olimpo, pascoli sassosi, 2300–2700 m, 6 August 2009, *F. Conti*, *D. Uzunov s.n.* (APP No. 55559); M. Nemercka a Diopaka, pendii rupestri e pascoli, 1995 m, 24 June 2015, *R. Di Pietro*, *D. Lakušić*, *N. Kuzmanović*, *A. Stinca*, *S. Đurović*, *I. Janković s.n.* (APP No. 57066); Tzoumerka, dal rifugio Melissourgoi verso lo Strogula, Kefalovryso, alla base della cascata, 1400 m, 17 July 2018, *F. Conti*, *V. Giacanelli s.n.* (APP Nos. 61315, 61316); Tzoumerka, poco sotto il rifugio Katarraktis, rupi e pendii rupestri, 1300 m, 18 July 2018, *F. Conti*, *V. Giacanelli s.n.* (APP No. 61372); Tzoumerka, dal rifugio in su lungo la strada (Katarraktis), 1900–2100 m, 18 July 2018, *F. Conti*, *V. Giacanelli s.n.* (APP No. 61390); Tzoumerka, dal rifugio Katarraktis verso la vetta Katafidi, 1700–1900 m, 18 July 2018, *F. Conti*, *V. Giacanelli s.n.* (APP Nos. 61407, 61408, 61419, 61427, 61430, 61443, 61460, 61463, 61466, 61469); Timfristos, 38.943753° N 21.824259° E, 1850–2300 m, 23 June 2023, *F. Conti*, *L. Bracchetti s.n.* (APP No. 70643); Kaliakouda, 38°.794662 21°.762204, rupi e pendii rupestri, 1950 m, 21 June 2023, *F. Conti*, *L. Bracchetti s.n.* (APP Nos. 71980, 71981, 71982, 71983, 71984, 71985).

***Leontodon hellenicus*** subsp. ***hellenicus***

GREECE: M. Chelidon, pendii rupestri, 21.685722 E 38.833621 N, 1800 m ca., 22 June 2023, *F. Conti*, *L. Bracchetti s.n.* (APP Nos. 70302, 70303, 70304); Kaliakouda, rupi e pendii rupestri, 21.762204 E 38.7944662 N, 1800 m ca., 21 June 2023, *F. Conti*, *L. Bracchetti s.n.* (APP Nos. 70305, 70306, 70307, 70308, 70309).

***Leontodon hellenicus*** subsp. ***valeriae***

GREECE: M.Tsoumerka-Pindo sud presso Melissourgoi, Vers: N, 1300 m ca., 14 June 1998, *F. Conti s.n.* (APP No. 47848); Tzoumerka. Dal rifugio Melissourgoi verso lo Strogula. Kefalovryso waterfall, alla base della cascata, 1400 m, 17 July 2018, *F. Conti*, *V. Giacanelli s.n.* (APP Nos. 61317, 61318, 61319, 61320, 61321)

Identification key to the studied taxa:

1. Outer phyllaries marginally ciliate with simple hair or glabrous ………………………………………… *L. crispus* subsp. *crispus* var. *crispus*

1. Outer phyllaries marginally ciliate with stellate hairs …………………… 2

2. Leaf hairs: most common number of distal rays, 3–4; number of hairs per 1 mm^2^ on leaf surface, 0–18 …………………………………………………………. 3

2. Leaf hairs: most common number of distal rays 5–8, number of hairs per 1 mm^2^ on leaf surface 12–62 ………………………………………………………… 4

3. Leaf hairs: most common number of distal rays (3–) 4, leaves with (0–) 5–13 (–18) hairs in 1 mm^2^ ………………………… *L. crispus* subsp. *asper*

3. Leaf hairs: most common number of distal rays 3 (–4), leaves with (0–) 3–6 (–8) hairs in 1 mm^2^ …………………………………… *L. crispus* var. *biscutellifolius*

4. Leaves with (18–) 28.7–38 (–62) hairs in 1 mm2, stalk length (0.09–) 0.1–0.14 (–0.2) [to maximally (0.12–) 0.14–0.2 (–0.24)], flowers length (14–) 15.6–19 (–22) mm, pappus length (6–) 7.0–8.85 (–10) ………… *L. hellenicus subsp. hellenicus*

4. Leaves with (12–) 17.2–27 (–41) hairs in 1 mm^2^; stalk length (0.1–) 0.13–0.27 [to maximally (0.16–) 0.24–0.38 (–0.46)], flower length (19–) 19.5–28.25 (–30) mm, pappus length (8.5–) 9.0–10.87 (–11.5) mm ……… *L. hellenicus* subsp. *valeriae*

## 5. Conclusions

The observed correlation of genetic and geographical patterns, which is parallelled by the results of the multivariate analysis based on morphological characters, argues for the distinctness of *crispus*/*biscutellifolius* on the one hand and *hellenicus*/*valeriae* on the other hand, with accessions of Albanian and Greece *asper* forming a connection via genetically and morphological intermediate forms. Taxonomically, this could be either accounted for by acknowledgement of the whole study group as a single species (*L. crispus*) with two subspecies (*crispus*/*biscutellifolius* vs. *hellenicus*/*valeriae*) and intermediate forms (*asper*). Owing to the present morphological results that show a closer relationship of *asper* accessions to *crispus*/*biscutellifolius* than to *hellenicus*/*valeriae* (see Figure 1 and Figure 2), however, we here propose to accept two independent species, i.e., *L. crispus* and *L. hellenicus*, and acknowledge the observed geographical and genetic correlations within these two entities on subspecies and variety level.

## Figures and Tables

**Table 1:** Morphological characters studied. Variables quantitative continuous (*) and quantitative discrete (**).

Variable
Plant height (cm) *
Leaf length (mm) *
Leaf width (mm) *
Number of teeth along each leaf side **
Width of leaf teeth (mm) *
Number of hairs per 1 mm^2^ on leaf surface **
Leaf hairs: most common number of distal rays **
Leaf hairs: minimum number of distal rays **
Leaf hairs: maximum number of distal rays **
Leaf hairs: stalk length (mm) *
Leaf hairs: maximum stalk length (mm) *
Leaf hairs: ray length (mm) *
Involucre: length (mm) *
Involucre: external bract length (mm) *
Fid hairs on external involucral bracts: number of rays **
Hairs on external involucral bracts: hair length (mm) *
Hairs on external and/or middle involucral bracts: length of simple moniliformis hairs (mm) *
Fid hairs on external involucral bract margins: number of rays **
Fid hairs on external involucral bract margins: stalk length (mm) *
Fid hairs on external involucral bract margins: ray length (mm) *
Fid hairs on middle involucral bract: number of rays **
Fid hairs on middle involucral bract: stalk length (mm) *
Fid hairs on middle involucral bract: ray length (mm) *
Flower length (mm) *
Achene: length (mm) *
Pappus: ray length (mm) *
Pappus: plume length (mm) *
Pappus: denticule length (mm) *

## Data Availability

The data presented in the current study are available within the article. Raw data of the AFLPseq fingerprinting were submitted to GenBank under the BioProject accession number PRJNA1315185.

## Appendix A

**Table A1:** Listing of all studied accessions; locality describes the collection site; date indicates when the sample was collected; longitude and latitude provide information about the coordinates; collector indicates who collected the sample and voucher indicates the exact sample or herbarium number.

Acc. n.	Taxon	Country	Data Label
A1302	*Leontodon crispus* subsp. *crispus*	Croatia	Sv. Jure-Lokve (Biokovo) 23 July 2001, *F. Conti*, *D. Tinti*, *M.E. Solic* (APP No. 47676)
A1303	*L. crispus* subsp. *crispus*	Croatia	Biokovo. Dal bivio per Vošac a Lokva 12 August 2018, *F. Conti*, *V. Giacanelli* (APP No. 58647)
A1304	*L. crispus* subsp. *crispus*	Croatia	Biokovo. Dal bivio per Vošac a Lokva 12 August 2018, *F. Conti*, *V. Giacanelli* (APP No. 58647)
A1305	*L. crispus* subsp. *crispus*	Croatia	Biokovo. Dal bivio per Vošac a Lokva 12 August 2018, *F. Conti*, *V. Giacanelli* (APP No. 58647)
A1310	*L. hellenicus* subsp. *hellenicus*	Greece	Mt. Chelidon, WGS84 21.685722 E 38.833621 N, 1800 m ca., pendii rupestri, 22 June 2023, *F. Conti*, *L. Bracchetti* (APP No. 70303)
A1311	*L. hellenicus* subsp. *hellenicus*	Greece	Kaliakouda, WGS84 21.762204 E 38.7944662 N, 1950 m, rupi e pendii rupestri, 21 Jne 2023, *F. Conti*, *L. Bracchetti* (APP No. 70305)
A1312	*L. hellenicus* subsp. *hellenicus*	Greece	Kaliakouda, WGS84 21.762204 E 38.7944662 N, 1950 m, rupi e pendii rupestri, 21 June 2023, *F. Conti*, *L. Bracchetti* (APP No. 70306)
A1313	*L. hellenicus* subsp. *hellenicus*	Greece	Kaliakouda, WGS84 21.762204 E 38.7944662 N, 1950 m, rupi e pendii rupestri, 21 June 2023, *F. Conti*, *L. Bracchetti* (APP No. 70308)
A1314	*L. hellenicus* subsp. *hellenicus*	Greece	Kaliakouda, WGS84 21.762204 E 38.7944662 N, 1950 m, rupi e pendii rupestri, 21 June 2023, *F. Conti*, *L. Bracchetti* (APP No. 70309)
A1320	*L. hellenicus* subsp. *valeriae*	Greece	Tzoumerka. Dal rifugio Melissourgoi verso lo Strogula. Kefalovryso waterfall alla base della cascata, 1400 m, 17 July 2018, *F. Conti*, *V. Giacanelli* (APP No. 61317)
A1321	*L. hellenicus* subsp. *valeriae*	Greece	Tzoumerka. Dal rifugio Melissourgoi verso lo Strogula. Kefalovryso waterfall alla base della cascata, 1400 m, 17 July 2018, *F. Conti*, *V. Giacanelli* (APP No. 61318)
A1322	*L. hellenicus* subsp. *valeriae*	Greece	Tzoumerka. Dal rifugio Melissourgoi verso lo Strogula. Kefalovryso waterfall alla base della cascata, 1400 m, 17 July 2018, *F. Conti*, *V. Giacanelli* (APP No. 61319)
A1323	*L. hellenicus* subsp. *valeriae*	Greece	Tzoumerka. Dal rifugio Melissourgoi verso lo Strogula. Kefalovryso waterfall alla base della cascata, 1400 m, 17 July 2018, *F. Conti*, *V. Giacanelli* (APP No. 61320)
A1324	*L. hellenicus* subsp. *valeriae*	Greece	Tzoumerka. Dal rifugio Melissourgoi verso lo Strogula. Kefalovryso waterfall alla base della cascata, 1400 m, 17 July 2018, *F. Conti*, *V. Giacanelli* (APP No. 61321)
A1339	*L. crispus* subsp. *asper*	Albania	Cika, vetta principale salendo dal passo Llogarasa 1700–2000 m, 9 July 2012, *F. Conti*, *M. Manilla* (APP No. 49333)
A1340	*L. crispus* subsp. *asper*	Greece	Tzoumerka. Dal rifugio Katarraktis verso la vetta Katafidi (Katarraktis), 1700–1900 m, 18 July 2018, *F. Conti*, *V. Giacanelli* (APP No. 61427)
A1341	*L. crispus* subsp. *asper*	Greece	Tzoumerka. Dal rifugio Katarraktis verso la vetta Katafidi (Katarraktis), 1700–1900 m, 18 July 2018, *F. Conti*, *V. Giacanelli* (APP No. 61460)
A1342	*L. crispus* subsp. *asper*	Greece	Tzoumerka. Dal rifugio Katarraktis verso la vetta Katafidi (Katarraktis), 1700–1900 m, 18 July 2018, *F. Conti*, *V. Giacanelli* (APP No. 61463)
A1343	*L. crispus* subsp. *asper*	Greece	Tzoumerka. Poco sotto il rifugio Katarraktis (Katarraktis), rupi e pendii rupestri, 1300 m, 18 July 2018, *F. Conti*, *V. Giacanelli* (APP No. 61372)
A1344	*L. crispus* subsp. *asper*	Greece	Kaliakouda, WGS84 21.762204 E 38.7944662 N, 1950 m, rupi e pendii rupestri, 21 June 2023, *F. Conti*, *L. Bracchetti* (APP No. 71983)
A1345	*L. crispus* subsp. *asper*	Greece	Kaliakouda, WGS84 21.762204 E 38.7944662 N, 1950 m, rupi e pendii rupestri, 21 June 2023, *F. Conti*, *L. Bracchetti* (APP No. 71985)
A1346	*L. crispus* var. *biscutellifolius*	Italy	Monte Maggiore versanti S, da Croce a San Salvatore (Rocchetta e Croce, CE), pascoli aridi, 570–857 m, 5 May 2017, *F. Conti*, *F. Bartolucci*, *R. Pennesi* (APP No. 57922)
A1348	*L. crispus* var. *biscutellifolius*	Italy	dal Rifugio del Monte al Vallone del Monte (FANO ADRIANO, TE), rupi (calcari marnosi e calcareniti) e pascoli su sedimenti morenici, 1615–2000 m, 19 June 2010, *F. Conti*, *F. Bartolucci*, *N. Ranalli* (APP No. 43736)
A1349	*L. crispus* var. *biscutellifolius*	Italy	Tra M. Irto e M. S. Nicola, Serra delle Gravare (OPI, AQ), 6 August 2010, *F. Conti*, *G. Serafini* (APP No. 48216)
A1350	*L. crispus* var. *biscutellifolius*	Italy	Faresano (Brienza, PZ), prato arido e gariga, 1050 m, 5 June 2013, *F. Conti*, *F. Bartolucci* (APP No. 52321)
A1351	*L. crispus* subsp. *asper*	Greece	Mt. Chelidon, WGS84 21.685722 E 38.833621 N, 1800 m ca., pendii rupestri, 22 June 2023, *F. Conti*, *L. Bracchetti* (APP No. 70979)
A1352	*L. crispus* subsp. *asper*	Greece	Mt. Chelidon, WGS84 21.685722 E 38.833621 N, 1800 m ca., pendii rupestri, 22 June 2023, *F. Conti*, *L. Bracchetti* (APP No. 71978)
A1353	*L. crispus* subsp. *asper*	Greece	Tzoumerka. Dal rifugio Katarraktis verso la vetta Katafidi (Katarraktis), 1700–1900 m, 18 July 2018, *F. Conti*, *V. Giacanelli* (APP Nos. 61407)
A1354	*L. crispus* subsp. *asper*	Greece	Tzoumerka. Dal rifugio Katarraktis verso la vetta Katafidi (Katarraktis), 1700–1900 m, 18 July 2018, *F. Conti*, *V. Giacanelli* (APP Nos. 61466)
A1355	*L. crispus* subsp. *crispus*	Croatia	Biokovo. Dal bivio per Vošac a Lokva 12 August 2018, *F. Conti*, *V. Giacanelli* (APP Nos. 58644)
A1356	*L. crispus* subsp. *crispus*	Croatia	Biokovo. Dal bivio per Vošac a Lokva 12 August 2018, *F. Conti*, *V. Giacanelli* (APP Nos. 58646)
A1357	*L. crispus* subsp. *crispus*	Croatia	Biokovo. Dal bivio per Vošac a Lokva 12 August 2018, *F. Conti*, *V. Giacanelli* (APP Nos. 58649)
A1358	*L. crispus* subsp. *crispus*	Croatia	Biokovo. Dal bivio per Vošac a Lokva 12 August 2018, *F. Conti*, *V. Giacanelli* (APP Nos. 58660)
A1438	*L. crispus* subsp. *crispus*	Italy	Monte Vaso (Chianni, PI), serpentino, 450–633 m, 23 May 2009, *F. Bartolucci, F. Conti* (APP No. 38404)
A1439	*L. crispus* subsp. *crispus*	Italy	Pesek (San Dorligo della Valle), presso Trieste 420 m, 31 May 2001, *F. Conti*, *L. Gubellini* (APP No. 47354)
A1441	*L. crispus* subsp. *crispus*	Italy	sotto case Baussuno (Cosio d’Arroscia, IM), 1090–1170 m, 29 June 2023, *F. Conti*, *F. Bartolucci* (APP No. 70052)
